# Psychosocial risks and their consequences on health and job satisfaction among Spanish police officers

**DOI:** 10.23938/ASSN.1058

**Published:** 2024-02-14

**Authors:** Ester Grau-Alberola, Antonio Berlanga Sánchez, Hugo Figueiredo-Ferraz

**Affiliations:** 1 Universidad Internacional de La Rioja (UNIR) School of Education Logroño Spain; 2 Police inspector Valencia Spain; 3 Valencian International University (VIU) School of Social Sciences and Law Valencia Spain; 4 University of Valencia Unidad de Investigación Psicosocial de la Conducta Organizacional (UNIPSICO) Valencia Spain

**Keywords:** Police officers, Occupational Stress, Occupational Risks, Occupational Health, Job Satisfaction, Policía, Estrés Laboral, Riesgos Laborales, Salud Laboral, Satisfacción en el Trabajo

## Abstract

**Background:**

Police officers are exposed to risk and violence, which makes their work highly stressful. According to the Job Demand-Resource model, the working conditions of police officers may affect their health and job satisfaction. The aims of this research were to evaluate the psychosocial factors at work in a sample of municipal police officers from the Valencian Community (Spain) to identify the prevalence of psychosocial risks and their consequences and analyse the influence of psychosocial factors (resource and demands) at work on job satisfaction and health.

**Methods:**

The *Unidad de Investigación Psicosocial de la Conducta Organizacional* (UNIPSICO) battery was used to evaluate the prevalence and distribution of different levels of risk and their consequences in police officers (n=103). The influence of psychosocial factors (resource and demands) at work on psychosomatic disorders and job satisfaction was examined using stepwise regression analysis.

**Results:**

All considered variables showed moderate levels of psychosocial risk for the whole sample. As for the consequences of psychosocial risks, moderate levels of psychosomatic disorders and job satisfaction were also observed. Perceived stress levels and organizational stressors have a negative effect on health and job satisfaction.

**Conclusions:**

To create positive conditions for the health and well-being of police officers, real commitment is required when evaluating psychosocial risks in local police organizations and the establishment of prevention and intervention measures where psychosocial risk factors are detected.

## Resumen

**Fundamento:**

La vida laboral del policía es estresante dada la exposición al riesgo y a la violencia en el trabajo. Según el Modelo de Demandas y Recursos laborales (DRL), las condiciones de trabajo de los policías pueden influir en la salud y la satisfacción laboral. Los objetivos de esta investigación fueron evaluar los factores psicosociales en el trabajo en una muestra de policías municipales de la Comunidad Valenciana (España), identificar la prevalencia de riesgos psicosociales y sus consecuencias, y analizar la influencia de los factores psicosociales (recursos y demandas) en el trabajo sobre la satisfacción laboral y la salud.

**Material y método:**

Se utilizó la Batería UNIPSICO para evaluar la prevalencia y la distribución de policías (n=103) en los diferentes niveles de riesgo y consecuencias. La influencia de los factores psicosociales (recursos y demandas) en el trabajo sobre los trastornos psicosomáticos y la satisfacción laboral se examinó mediante un análisis de regresión por pasos.

**Resultados:**

Se obtuvieron valores moderados de riesgo psicosocial en todas las variables para toda la muestra. Para las consecuencias de los riesgos psicosociales también se obtuvieron niveles moderados de trastornos psicosomáticos y satisfacción laboral. Los niveles de estrés percibidos y los estresores organizacionales tienen un efecto negativo sobre la salud y la satisfacción laboral.

**Conclusión:**

Para facilitar condiciones positivas para la salud y el bienestar de los policías, se requiere un compromiso real para la evaluación de riesgos psicosociales en las organizaciones policiales locales y para la adopción de medidas de prevención e intervención, donde se detecten factores de riesgo psicosocial.

## INTRODUCTION

Police officers are exposed to risk and violence, which makes their work highly stressful^1^. Police work often involves confronting situations that require an immediate response under unpredictable and uncertain conditions^2^; it also implies a high degree of social interaction with a high level of emotional involvement from the worker. The characteristics of the organization that defines the professional performance of the police are complex, involving tasks with a wide range of responsibility and roles that are difficult to fulfil. Thus, improving organizational behaviour, rather than personal characteristics, may be more efficient to improve overall police performance^3^. The working conditions of police officers may influence health^4^, performance, and job satisfaction^5^.

Occupational health in Spain aims to achieve the highest degree possible of physical, mental, and social welfare of the workers with regard to risks and work environment. In Spain, Prevention of Workplace Risks Law 31/1995 regulates occupational risk prevention^6^; article 3.2 of this law indicates that it is fully applicable to police forces, with some exceptions. Nevertheless, it does not exempt ensuring the safety and health of the workers, especially health surveillance and psychosocial risk assessment.

In the “job demands” model, Karasek^7^ states that psychological strain occurs when demands (e.g., work overload) exceed personal resources (e.g., job decision latitude, autonomy). However, the Job Demand-Resource model (JD-R) highlights that any demand and resource may affect the health and well-being of the employee^8^. According to latter model, job demands represent components of the work environment that require employees to exert continual physical and/or mental effort. Job resources (grouped into physical, social, psychological, and/or organizational components) may reduce job demands and the resultant physiological and psychological reactions^9^.

Numerous psychosocial factors in the police officer working environment involve interactions between stressors, coping strategies, and culture^10^. Thus, it is possible to identify psychosocial resource factors and psychosocial demands on police, at work^11^.

Lucero-Moreno et al.^12^ point out that in studies on police work in Spain, occupational stress mainly focus on burnout, which makes it difficult to link the results to the stressors. Health problems are most frequently mentioned among the negative effects of job stressors, which have been observed in police officers and are significantly associated with high levels of job-related stress^13^.

The aims of this study were to evaluate the psychosocial factors at work in a sample of municipal police officers from the Valencian Community (Spain) to identify the prevalence of psychosocial risks and their consequences (on job satisfaction and health) and analyse the influence of psychosocial resource factors and psychosocial demands on job satisfaction and health.

## MATERIALS AND METHODS

Cross-sectional, non-randomized study of the psychosocial risks in municipal police officers in Valencia, Valencian Community (Spain), carried out in October 2019. 

A self-administered questionnaire was sent by mail to Valencian municipal police officers. They were informed about the purpose of the study and signed an informed consent of their voluntary participation. 

The Human Research Ethics Committee of the University of Valencia (Spain) approved the study (Resolution_1657624953552_au2k_1057_2082431_1); anonymity was guaranteed to all participants^14^. 

The evaluation of the psychosocial factors (demands, resources, and their consequences) was carried out using the Spanish version of the *UNIPSICO* battery^15^^-^^17^. This questionnaire evaluates the psychosocial situation of a group of workers with the aim of recommending preventative and intervention measures to improve their quality of working life. Items were based on a 5-point frequency scale: never=0, rarely/few times a year=1; sometimes/a few times a month=2; frequently/a few times a week=3, quite frequently/every day=4.

- The six psychosocial demand factors evaluated were workload (6 items) (α = 0.64), interpersonal conflicts (6 items) (α = 0.86), role conflict (5 items) (α = 0.76), role ambiguity (5 items, inversed for analyses) (α = 0.77), work-family conflict (6 items) (α = 0.87), and inequity in social exchanges (5 items) (α = 0.80); high scores indicated deterioration of the working conditions.
- The four psychosocial resource factors evaluated were autonomy (5 items) (α = 0.77), social support at work (6 items) (α = 0.87), availability of resources (7 items) (α = 0.84), and feedback (8 items) (α = 0.84); high scores indicated satisfaction with the working conditions. In addition, leadership style was evaluated (4 items for transformational leadership and 4 items for *laissez-faire* leadership) (α = 0.88)*.*
- The consequences of psychosocial risks evaluated were psychosomatic disorders (13 items) (headaches, musculoskeletal pain, sleep quality, anxiety, and sickness) (α = 0.89) and job satisfaction (6 items) (α = 0.79); high scores indicated high frequency of psychosomatic disorders and that the worker was satisfied with his/her working conditions.

In addition, sociodemographic (age, sex, children in charge, seniors in charge, marital status, and university studies) and socio-occupational variables (years as police worker, years in the job, police rank and functions) were analysed. Frequency and percentage were calculated for categorical variables, and mean and standard deviation (SD) for the quantitative ones.

To categorize the exposure to psychosocial factors and their consequences, percentiles 33 and 66 (P_33_, P_66_) were used; scores ≤ P_33_ = low level; scores > P_33_ and < P_66_ = medium, and scores ≥ P_66_ = high level.

A two-step linear regression analysis (stepwise method) was performed to determine what percentages of variance in psychosomatic disorders and job satisfaction were explained by the independent variables (resource factors and demand factors). Four models were obtained to evaluate the influence of resource factors and demand factors separately on the evaluated consequences. For demand factors, the variables sex and age were introduced in the first step, and the six demand variables and *laissez-faire* leadership style in the second one. For resource factors, sex and age were introduced in the first step, and the four resource variables and transformational leadership in the second one. All analyses were conducted using SPSS v26.

## RESULTS

Overall, the response rate was 7%; 103 questionnaires (of the 1,469 sent) were returned and correctly completed. Most study participants were male (86.4%), mean age 41.45 years (SD: 7.55, range: 24-60), with university studies, and had a stable partner (Table 1).

Regarding the type of job, most participants worked on patrol (citizen security functions, wide area of action in relation to crime problems), while the remaining performed administrative duties (management of reports, crime reports, letters to the courts) or were proximity police (functions with the community, action in the same area, direct contact with citizens) (Table 1).

**Table 1:** Characteristics of the study population

Variables	n (%)
Children in charge	74 (71.8)
Seniors in charge	15 (14.6)
*Marital status*	
Single	21 (20.4%)
Partner	64 (62.1%)
Divorced	18 (17.5%)
University studies	70 (68.0)
Years in the job*	7.77 (7.40)
Years in the profession*	16.45(9.12)
*Police functions*	
On patrol	74 (71.8%)
Administrative duties	16 (15.5%)
Proximity police	13 (12.6%)
*Police rank*	
Agents	59 (57.3)
Superior	44 (42.7)

*: mean (standard deviation).

Moderate scores were obtained for all variables of the UNIPSICO bettery (Table 2).

**Table 2:** Descriptive statistics and prevalence rates of psychosocial factors (demands, resources, and their consequences) from the Spanish version of the UNIPSICO battery

Psychosocial factors	Descriptive statistics							Prevalence rates (%)		
	Mean	SD	95%CI	Min	Max	P_33_	P_66_	L	M	H
*Demands*										
Workload	2.15	0.60	2.28-2.52	1.00	3.83	1.83	2.33	37.9	32.0	30.1
Interpersonal conflicts	1.07	0.73	0.92-1.21	0	3.67	0.83	1.17	44.7	27.1	28.2
Role conflict	1.47	0.78	1.31-1.62	0	3.60	1.00	1.80	37.9	33.0	29.1
Role ambiguity	3.13	0.62	3.01-3.25	1.20	4.00	3.00	3.40	45.6	22.4	32.0
Work-family conflict	1.08	0.85	0.91-1.24	0	3.33	0.50	1.17	34.0	34.9	31.1
Inequity	2.02	0.86	1.85-2.18	0.20	4.00	1.60	2.40	35.0	36.8	28.2
*Resources*										
Autonomy	2.62	0.64	2.49-2.74	0.40	4.00	2.40	3.00	38.8	35.0	26.2
Social support	2.85	0.82	2.69-3.01	0.50	4.00	2.50	3.33	35.9	32.1	32.0
Resources	2.06	0.72	1.91-2.19	0.29	3.57	1.71	2.52	35.0	40.7	24.3
Feedback	2.03	0.80	1.87-2.18	0.25	4.00	1.67	2.25	33.0	35.0	32.0
Transformational	2.22	1.01	2.02-2.41	0	4.00	1.83	2.50	33.0	34.0	33.0
*Laissez-Faire*	1.46	0.78	1.30-1.60	0	3.00	1.00	1.67	36.9	31.1	32.0
*Consequences*										
Psychosomatic disorders	0.97	0.75	0.82-1.11	0	3.33	0.56	1.11	35.0	30.0	35.0
Job satisfaction	2.30	0.70	2.16-2.43	0.50	3.83	2.00	2.67	36.9	34.9	28.2

SD: standard deviation; CI: confidence interval; Min: minimum; Max: máximum; P: percentile; L: low; M: medium; H: high.

As for psychosocial demand factors, the largest percentage of participants with high levels of psychosocial risk was seen for the role ambiguity variable (measured as role clarity) (45.6%). For this variable, more than a third of the participants perceived high levels of psychosocial risk.

Regarding psychosocial resource factors, the largest percentages of risk (low scores) were found for the variables autonomy (38.8%), social support at work (35.9%), and availability of resources (35%); more than a third of the participants perceived high levels of psychosocial risk.

**Figure 1:**
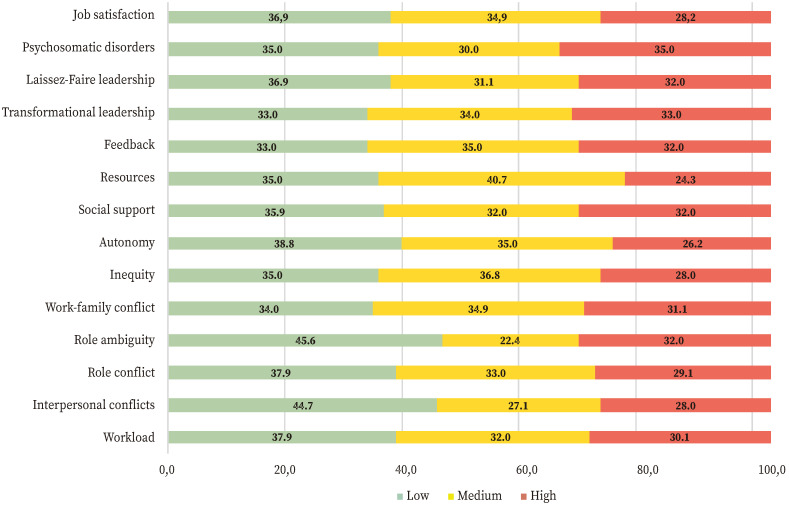
Prevalence rates of psychosocial factors from the Spanish version of the UNIPSICO battery (%).

Considering the consequences of psychosocial risks, 36.9% of study participants expressed job dissatisfaction in comparison with 28.2% who responded they were satisfied with their working conditions (Table 2, Fig. 1).

Finally, 36 of the participants (35%) stated they had work-related psychosomatic disorders. Likewise, 12 (11.7%) answered that over the past year they had taken medication frequently or very frequently to treat work-related psychosomatic health and 5 (4.8%) had frequently or very frequently needed the support of a specialist to overcome work-related personal crises. In addition, 19 participants (17.8%) had increased alcohol use and 15 (14.6%) tobacco use, both associated to work-related issues.

The next step in the evaluation of psychosocial risks was to analyse their influence on psychosomatic disorders and job satisfaction. 

Regarding the prediction of psychosomatic disorders, the results showed that *age* (Model 1) explained 6% of the variance and *availability of resources* (Model 2) explained an additional 12% of the variance. When *social support* was added (Model 3), it accounted for an additional 5% of the variance. In total, these resource factors explained 23% of the variance (Table 3).

In relation to demand factors, the results showed that for psychosomatic disorders, *age* (Model 1) explained 6% of the variance and *interpersonal conflicts* (Model 2) explained an additional 32% of the variance. In total, these demand factors explained 38% of the variance (Table 3).

**Table 3:** Standardized regression coefficients (ß) for demographic variables, job resources, and job demands as predictors of psychosomatic disorders and job satisfaction

		Model			
		1	2	3	4
*Psychosomatic disorders*					
*Job resources*					
Resources			-0.34 (<0.001)	-0.25 (<0.01)	
Social support				-0.26 (<0.01)	
Age (control variable)		0.24 (<0.05)	0.21 (<0.05)	0.12	
	R^2^	0.06	0.17	0.22	
	ΔR^2^	0.06	0.12	0.05	
	F (p) for change in R^2^	5.90 (<0.05)	13.64 (<0.001)	6.43 (<0.05)	
*Job demands*					
Interpersonal conflicts			0.62 (<0.001)		
Age (control variable)		0.24 (<0.05)	-0.03		
	R^2^	0.06	0.38		
	ΔR^2^	0.06	0.32		
	F (p) for change in R^2^	5.90 (<0.05)	50.86 (<0.001)		
*Job satisfaction*					
*Job resources*					
Resources			0.52 (<0.001)	0.44 (<0.001)	0.38 (<0.001)
Transformational leadership			0.38 (<0.001)	0.25 (<0.01)	
Social support					0.25 (<0.05)
Age (control variable)		-0.30 (<0.001)	-0.26 (<0.001)	-0.18 (<0.05)	-0.12
	R^2^	0.09	0.36	0.49	0.52
	ΔR^2^	0.09	0.27	0.13	0.03
	F for change in R^2^	9.72 (<0.01)	41.96 (<0.001)	24.94 (<0.001)	6.65 (<0.05)
*Job demands*					
Role ambiguity			0.44 (<0.001)	0.32 (<0.001)	
Inequity				-0.31 (<0.01)	
Age (control variable)		-0.30 (<0.01)	-0.25 (<0.01)	-0.19 (<0.05)	
	R^2^	0.09	0.28	0.36	
	ΔR^2^	0.09	0.19	0.08	
	F (p) for change in R^2^	9.72 (<0.01)	24.34 (<0.001)	12.07 (<0.001)	

Regarding the prediction of job satisfaction, the results showed that *age* (Model 1) explained 9% of the variance and *availability of resources* (Model 2) explained an additional 27% of the variance. When *transformational leadership* was added (Model 3), it accounted for an additional 13% of the variance. Finally, *social support* explained an additional 3% of the variance. In total, these resource factors explained 52% of the variance (Table 3).

As for demand factors, the results showed that for job satisfaction, *age* (Model 1) explained 9% of the variance and *role ambiguity* (measured as *role clarity*) (Model 2) explained an additional 19% of the variance. When *inequity in social exchanges* was added (Model 3), it accounted for an additional 8% of the variance. In total, these demand factors explained 36% of the variance for job satisfaction (Table 3).

Other variables evaluated, as either demand factors or resource factors, were not significant predictors leading to psychosomatic disorders or job dissatisfaction.

## DISCUSSION

In this study, we examine the psychosocial characteristics of police work, the prevalence of demand and resource factors on workers, and the influence these factors have on job satisfaction and psychosomatic disorders. 

Overall, we observe moderate stress levels. This is in agreement with the results of a study carried out with Portuguese police officers that reports moderate levels of operational stress, same as the consequences^18^. Likewise, moderate levels of psychosocial risks were reported in 26 out of 29 studies in a systematic review with police officers^1^. However, in Spain, a study carried out with local police officers in Madrid communicated high levels of stress regardless of their occupational rank, sex, age, or type of shift; the participants perceived the evaluated psychosocial risk factors adversely^12^. These results may be explained considering the peculiarities of police work in each city. Work in large cities may increase stress among police officers due to their frequent exposure to critical and extremely stressful situations^1^. In addition, the use of different measures should be considered when comparing the results^1^.

As for predicting psychosomatic disorders, our results show that the availability of resources and perception of social support are key variables with a positive impact on health. On the other hand, interpersonal conflicts may cause psychosomatic disorders in the study sample. In addition, our results show that culture and work climate, leadership style developed within the police organization, and perceived social support are important factors associated to job satisfaction.

The results obtained are in line with other studies involving police officers. Arial et al.^19^, point out that certain work stressors, such as the lack of support from superiors and/or from the organization, associate with psychiatric symptoms. A systematic review showed that the organizational stressors most often associated with mental health outcomes include the lack of support^20^. The authors point out that there is a significant association between organizational stressors and their consequences, which include psychiatric symptoms and psychological distress. Our results are in line with other studies carried out with Spanish police officers. A study with 182 police officers found that social support and control were significant predictors of job satisfaction^21^.

The lack of resources associates to worse perceived health; thus, practical implications include specifying the resources needed in every situation^22^. The lack of personal protective equipment and police protection implies overexposure to risks. As for the working conditions, organizational issues concerning work, shifts, level of insecurity, and temporary work are key. As noted in the meta-analysis^1^, negative working conditions affect both mental and physical health, resulting in stress-related disorders and depression. 

Moreover, Swid^23^ conducted a study with 124 police officers in the United States and found that leadership style (transformational leadership) was a significant predictor of job satisfaction.

With respect to interpersonal factors (due to the nature of the job), police officers have frequent interpersonal interactions with the public, colleagues, and the police organization as a whole. These interactions may be a source of conflict and consequently affect health. This can be explained by the fact that police officers - at individual level - do not necessarily have the appropriate skills that may consequently lead to the development of negative emotions and affect their mental health^24^. Baka^25^ concluded that interpersonal conflicts are a significant predictor of health complaints (e.g., high levels of depression).

In our study sample, role ambiguity and inequity have a negative impact on job satisfaction. This finding confirms that certain organizational stressors are important predictors of a low rate of job satisfaction in Spanish local police officers. Role ambiguity has a negative influence on the development of satisfaction; thus, it is necessary to set clear expectations about the roles in the organization. Rhodes^26^ carried out a research among school resource officers and patrol officers and found that role ambiguity was a significant negative predictor of job satisfaction. Our results are in line with other studies in which the authors conclude that perceived stress levels and organizational stressors have a negative effect on job satisfaction^27^.

Violanti et al.^28^ point out the need of further studies assessing the organizational impact of stressors on the health of police officers. 

There are two key interventional solutions to reduce work stress and avoid negative consequences for workers: prevention and adequate training. Learning to cope with stress may help prevent the occurrence of more serious consequences, such as burnout and acute health problems. To achieve a positive outcome, it would be advisable to develop training programs that include emotional and cognitive-behavioural elements and social support, and to analyse its effectiveness in reducing stress levels and other work-related health problems^29^.

Among the limitations of the study, the sincerity and motivation of the participants in completing the questionnaire should considered. Secondly, the external validity of our results; the data were not collected randomly and participation in the study was voluntary. This is a cross-sectional study, thus, the direction of the identified risk factors cannot be established. For this reason, further studies with this population should be carried out and consider a larger sample size in order to analyse the differences based on sex and occupational rank^1^.

Analysis of the psychosocial characteristics of police work shows that it is a complex occupation subjected to multiple internal and external variables that involve psychosocial risk factors with psychological, physical, and social consequences^1^^,^^4^^,^^30^.

In line with this, Galanis et al.^1^ suggest further well-designed studies with a more rigorous methodology to better identify the real stress factors in the working lives of the police.

In conclusion, police officers need a safe and supportive environment, particularly because their job demands are highly stressful. Training programs can improve their perception of stress by promoting the learning of coping strategies and self-management, thereby reducing the negative consequences on physical and psychological health. This way, the individual’s perception of demands would be in line with the commitment of the organization and may help improve individual and collective satisfaction. From an integrative perspective, multiple components are developed and interrelated in the organization. Thus, in order to improve police well-being, it is necessary to inform and train to generate group cohesion and organizational commitment.

## References

[1] Galanis P, Fragkou D, Katsoulas TA (2021). Risk factors for stress among police officers: A systematic literature review. Work.

[2] CIESLAK I, Kielan A, Olejniczak D, Panczyk M, Jaworski M, Galazkowski R (2020). Stress at work: The case of municipal police officers. Work.

[3] Jang C (2020). The effect of police officers' participatory decision-making, supervisory quality and attitude and organizational justice on job satisfaction: focused on mediating effect of work-life conflict. Int J Adv Cult Technol.

[4] Magnavita N, Capitanelli I, Garbarino S, Pira E (2018). Work-related stress as a cardiovascular risk factor in police officers: a systematic review of evidence. Int Arch Occup Environ Health.

[5] Brady PQ, King WR (2018). Brass satisfaction: identifying the personal and work-related factors associated with job satisfaction among police chiefs. Police Q.

[6] Jefatura del Estado (1995). Ley 31/1995, de 8 de noviembre, de Prevención de Riesgos Laborales. Boletín Oficial del Estado.

[7] Karasek RA (1979). Job demands, job decision latitude, and mental strain: implications for job design. Admin Sci Q.

[8] Bakker AB, De Vries JD (2021). Job demands-resources theory and self-regulation: new explanations and remedies for job burnout. Anxiety Stress Coping.

[9] Bakker AB, Demerouti E (2007). The job demands-resources model: state of the art. J Manag Psychol.

[10] Gutschmidt D, Vera A (2022). Organizational culture, stress, and coping strategies in the police: an empirical investigation. Police Pract Res.

[11] Frank J, Lambert EG, Qureshi H (2017). Examining police officer work stress using the job demands-resources model. J Contemp Crim Justice.

[12] Luceño ML, García AY, Talavera VB, Martín GJ (2016). Stress in Spanish police force depending on occupational rank, sex, age and work-shift. Psicothema.

[13] Chan JF, Andersen JP (2020). Influence of organizational stress on reported depressive symptoms among police. Occup Med (Lond).

[14] Berlanga Sánchez A (2022). Mediación Policial: diseño curricular, formación y buenas prácticas en métodos alternativos de resolución de conflictos y gestión emocional.

[15] Gil-Monte PR, López-Vílchez J, Llorca-Rubio JL, Sánchez Piernas J (2016). Prevalencia de riesgos psicosociales en el personal de la administración de justicia de la comunidad de Valencia (España). Lib Rev Peru Psicol.

[16] Grau-Alberola E, Gil-Monte PR, Figueiredo-Ferraz H (2016). Influence of work-family conflict on health: A gender perspective. Int J Psychol.

[17] Grau-Alberola E, Figueiredo-Ferraz H, López-Vílchez JJ, Gil-Monte PR (2022). The healthy management: the moderator role of transformational leadership on health workers. An Psicol.

[18] Queirós C, Passos F, Bártolo A, Marques AJ, Da Silva CF, Pereira A (2020). Burnout and stress measurement in police officers: literature review and a study with the operational police stress questionnaire. Front Psychol.

[19] Arial M, Gonik V, Wild P, Danuser B (2010). Association of work-related chronic stressors and psychiatric symptoms in a Swiss sample of police officers: a cross sectional questionnaire study. Int Arch Occup Environ Health.

[20] Purba A, Demou E (2019). The relationship between organizational stressors and mental wellbeing within police officers: a systematic review. BMC Public Health.

[21] Marcos A, García-Ael C, Topa G (2020). The influence of work resources, demands, and organizational culture on job satisfaction, organizational commitment, and citizenship behaviors of spanish police officers. Int J Environ Res Public Health.

[22] 22. Tuckey MR, Chrisopoulos S, Dollard MF. Job demands, resource deficiencies, and workplace harassment: Evidence for micro-level effects. Int J Stress Manage 2012; 19(4): 292-310. Doi: 10.1037/a0030317

[23] Swid A (2014). Police members perception of their leaders' leadership style and its implications. Policing.

[24] Andersen JP, Gustafsberg H (2016). A training method to improve police use of force decision making: a randomized controlled trial. SAGE Open.

[25] Baka L (2015). The effects of job demands on mental and physical health in the group of police officers. Testing the mediating role of job burnout. Studia Psychol.

[26] Rhodes TN (2015). Officers and school settings examining the influence of the school environment on officer roles and job satisfaction. Police Q.

[27] Alexopoulos EC, Palatsidi V, Tigani X, Darviri C (2014). Exploring stress levels, job satisfaction, and quality of life in a sample of police officers in Greece. Saf Health Work.

[28] Violanti JM, Charles LE, McCanlies E, Hartley TA, Baughman P, Andrew ME (2017). Police stressors and health a state-of-the-art review. Policing.

[29] Figueiredo-Ferraz H, Grau-Alberola E, Gil-Monte PR (2019). Prevención y tratamiento del síndrome de quemarse por el trabajo.

[30] Goth J, Pfeffer J, Zenios SA (2015). Workplace stressors and health outcomes: health policy for the workplace. Behav Sci Policy.

